# Single-shot X-ray imaging of two-dimensional strain fields in colloidal crystals

**DOI:** 10.1107/S2052252524012521

**Published:** 2025-02-11

**Authors:** Jiecheng Diao, Zichen Gao, Jiadong Fan, Yajun Tong, Hang Ren, Yonggan Nie, Ian Robinson, Huaidong Jiang

**Affiliations:** a Center for Transformative Science ShanghaiTech University Shanghai201210 People’s Republic of China; b London Centre for Nanotechnology University College London LondonWC1E 6BT United Kingdom; Harima Institute, Japan

**Keywords:** X-ray free electron lasers, XFELs, Bragg coherent diffraction imaging, BCDI, colloidal crystals, coherent X-ray diffractive imaging, CXDI, diffract-then-destroy, inorganic materials

## Abstract

We used a soft X-ray free-electron laser and the Bragg coherent diffraction imaging method to characterize the defect structure of colloidal crystals. The single-shot X-ray pulse allowed us to reach four powder rings and measured all six reflections of the hexagonal lattice. We reproduced the static shape of the 2D crystal and mapped out the 2D strain tensors inside the crystal. The observed defect structures agreed with electron microscope images of similar colloidal samples.

## Abstract 2

We used the Bragg coherent diffraction imaging method at the Coherent Scattering and Imaging endstation of the Shanghai Soft X-ray Free Electron Laser Facility to characterize colloidal crystals. This method successfully reproduced the static shape of crystals and we observed the defect structure of colloidal samples.

## 1. Introduction

Colloidal crystals have emerged as a practical material with tunable mechanical, electronic and photonic properties. They are usually made from the bottom-up self-assembly of relatively monodispersed colloidal particles into periodically arranged uniform blocks, which allow precise and relatively easy control in the size, species and stoichiometry of the constituents (Shevchenko *et al.*, 2006 ▸; Udayabhaskararao *et al.*, 2017 ▸; Macfarlane *et al.*, 2013 ▸; Cherniukh *et al.*, 2021 ▸). Therefore, diverse structure ordering into assemblies with distinct properties could be prepared in a controllable manner. Planar, 2D colloidal crystals have also been used as model systems for studying phonon dispersion and topological phase transitions in 2D (Murray & Winkle, 1987 ▸; Murray & Wenk, 1989 ▸; Hirth *et al.*, 2020 ▸). For example, the Kosterlitz–Thouless–Halperin–Nelson–Young theory predicts the existence and thermal excitation of disclinations and the existence of hexatic phases, which guide our understanding of 2D solid melting (Kosterlitz & Thouless, 1973 ▸; Halperin & Nelson, 1978 ▸; Young, 1979 ▸). This work led to the 2016 Nobel Prize in physics.

Bragg coherent diffraction imaging (BCDI) is a coherence-based X-ray imaging method, which holds unique sensitivity to crystal defects and has profound applications in ferroelectrics, batteries and photovoltaics (Robinson & Harder, 2009 ▸; Ulvestad *et al.*, 2015 ▸; Diao *et al.*, 2020*a* ▸; Orr *et al.*, 2023 ▸). By looking at a structure via the diffraction peaks, BCDI has inherent sensitivity to crystal strains. The method has been applied to Au and BaTiO_3_ nanocrystals to study the continuous lattice dynamics on picosecond timescales (Clark *et al.*, 2013 ▸; Diao *et al.*, 2020*b* ▸). For the colloidal crystal system, plane-wave coherent diffraction imaging has been performed at both synchrotrons and hard X-ray free-electron lasers (XFELs) for structural characterization and femtosecond laser response (Shabalin *et al.*, 2016 ▸; Carnis *et al.*, 2021 ▸; Mukharamova *et al.*, 2020 ▸). However, it lacks a direct probe of the lattice displacement. With the advent of the soft XFEL, it has become possible to consider studying the melting of topological defects in colloidal crystals on the ultrafast timescale and with nanometre resolution. In this work, a single-shot BCDI experiment was performed to map out the static 2D structure of an Au colloidal crystal.

## 2. Defects in Au colloidal crystals

Au nanoparticles from NanoSeedz (model NS-40) with a diameter of 40 ± 3 nm were used for the experiment. The Au nanoparticles were coated with cetyltri­methyl­ammonium bromide and dispersed in water. Without hy­droxy­lation, 50 nm-thick Si_3_N_4_ membranes were used as the supporting substrate and heated to 70°C. 40 µl of Au nanoparticle aqueous solution was then dropped onto the Si_3_N_4_ film, where the nanoparticles formed small agglomerates after water evaporation. In the sample preparation test, we found that choosing a high concentration of Au particles (OD = 5) and heating to evaporate the solvent quickly would be conducive to form small clusters. If the sample solution concentration is diluted by more than ten times, the Au particles will become more scattered and may not assemble. If the sample solution is not heated, the Au particles will assemble at the position of final evaporation of the solvent. The assembly area is too large and is not suitable for BCDI.

**Figure 1:**
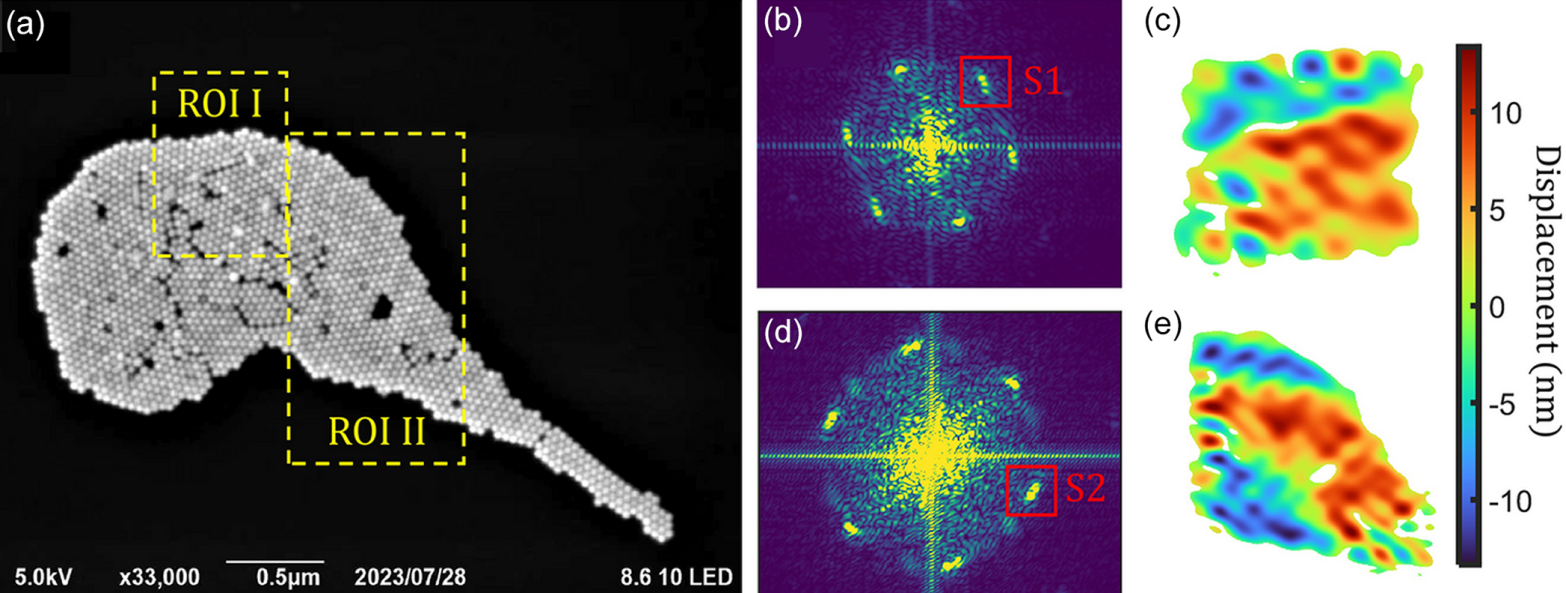
GPA of the SEM micrograph. (*a*) Typical SEM micrograph of the prepared Au colloidal crystals. Two ROIs are selected and indicated by the yellow-dashed lines. (*b*) and (*d*) Simulated diffractions of ROI I and ROI II. (*c*) and (*e*) Fourier transform of the diffraction peak S1 and S2 marked by red boxes in (*b*) and (*d*), respectively.

To obtain some pre-knowledge of the as-prepared Au colloidal crystals, a scanning electron microscope (SEM) was used to visualize the Au colloidal crystals before the diffraction experiment. Fig. 1 ▸(*a*) shows a micrograph containing a cluster of densely packed colloidal crystals. Cracks and voids are scattered all over the cluster, together with different local orientations of the lattice. The Fourier transform of the whole cluster provides a diffuse powder ring, indicating a multi-crystalline structure. Two regions of interest (ROIs) were selected, shown as the area surrounded by the yellow-dashed boxes in Fig. 1 ▸(*a*). These two ROIs were cropped out and Fourier transformed to generate the diffraction pattern individually, shown in Figs. 1 ▸(*b*) and 1 ▸(*d*). Both diffraction patterns have sixfold symmetric diffraction peaks, indicating a locally hexagonal close-packed lattice. Only one set of sixfold peaks could be observed, indicating that the atoms in the regions largely hold one uniform orientation. To map the local displacement field quantitatively, geometric phase analysis (GPA) was carried out. GPA was proposed by Hÿtch *et al.* (1998 ▸, 2003 ▸) and has since been developed as a powerful tool to analyse the deformation and strain in high-resolution transmission electron microscopy images. In Figs. 1 ▸(*b*) and 1 ▸(*d*), two regions indicated by red boxes containing diffraction peaks S1 and S2 were selected and Fourier transformed back to real space, as shown in Figs. 1 ▸(*c*) and 1 ▸(*e*). In both cases, the reconstructed images reproduce the shape of the ROI well. The displacement fields inside the colloidal crystals were clustered together and formed domains with similar local displacement. The domain walls were clear, indicating symmetry breaking of the lattice. Notably, not all the defects in the SEM micrograph in Fig. 1 ▸(*a*) show up in the GPA images in Figs. 1 ▸(*c*) and 1 ▸(*e*). This is because the images show only the projected displacement field corresponding to the **Q** direction of the selected peaks.

## 3. Single-shot X-ray diffraction of colloidal crystals

**Figure 2:**
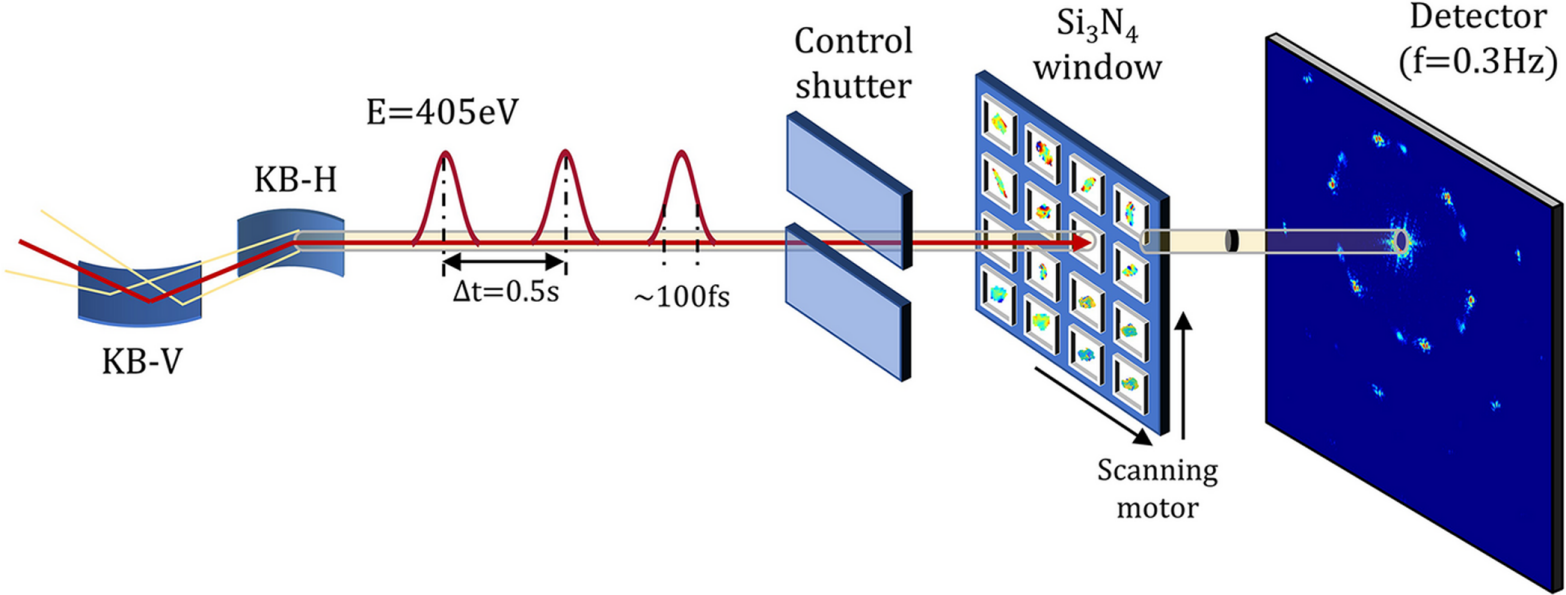
The X-ray diffraction setup at the CSI beamline of SXFEL.

After checking with SEM, the single-shot X-ray imaging was carried out on the same 2D Au colloidal crystal samples. The coherent diffraction experiment was performed at the Coherent Scattering and Imaging (CSI) endstation, which is one of the five beamlines currently in operation at the Shanghai Soft X-ray Free Electron Laser Facility (SXFEL). Fig. 2 ▸ shows the setup of the diffraction experiment. The X-ray energy was fixed at 406.5 eV in this experiment, where the pulse energy was estimated to be around 200 µJ (Gao *et al.*, 2024 ▸). The pulse repetition rate was set at 2 Hz with a duration of 100 fs. The X-ray pulses were focused down to around 3 µm at the sample position by a pair of Kirkpatrick–Baez mirrors (Gao *et al.*, 2023 ▸). The Si_3_N_4_ window with deposited Au colloidal crystal samples was placed vertically to enable a Laue diffraction geometry. A 16M charged-coupled device (CCD) PI-MTE3 detector with a pixel size of 15 µm was positioned 166.1 mm downstream from the sample stage, operating at a frequency of 0.3 Hz (Gao *et al.*, 2024 ▸). The mismatch in frequency coupling of the X-ray pulse and CCD detector was corrected by introducing a control shutter before the sample stage. Along with two scanning motors moving perpendicular to the X-ray beam, this enabled a successive scanning of all the windows in a step-and-shoot mode, in which each single-shot X-ray pulse hit one sample window to give one full-field diffraction pattern measured by the detector. Due to the destructive nature of the XFEL single pulses on the sample, each signal collection resulted in irreversible damage to the sampled position (Neutze *et al.*, 2000 ▸). Once the pattern from a single-pulse diffraction was recorded, a background-subtraction procedure was carried out, which includes a dark-field correction and a threshold filtering.

**Figure 3:**
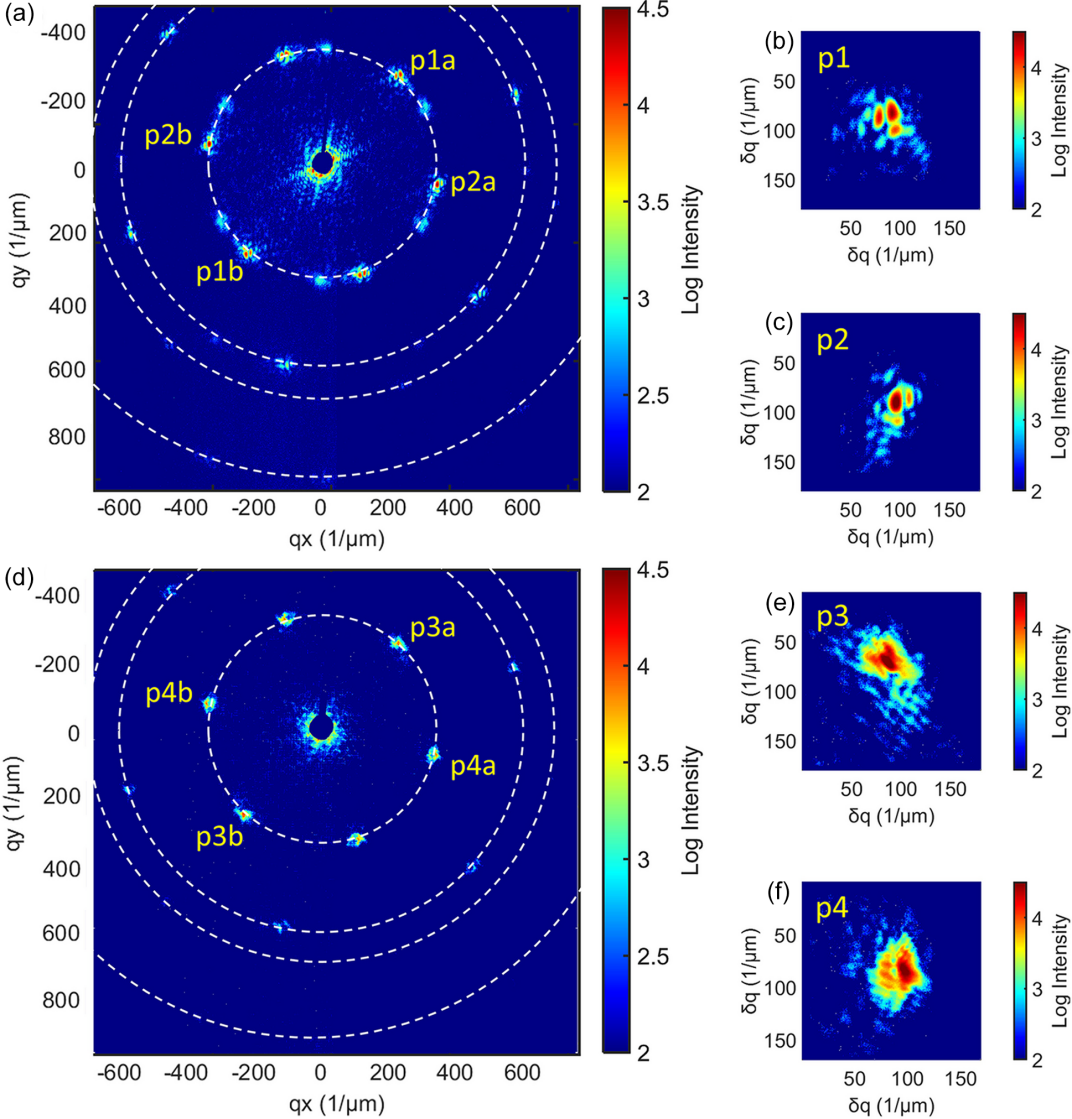
Diffraction pattern from the single-pulse coherent X-ray scattering from a 2D colloidal Au crystal. (*a*) and (*d*) Two full-field detector images on the 16M CCD detector, which show hexagonal diffraction peaks on four powder rings. The white dashed circles serve to guide the eye. (*b*) An intensity-enhanced diffraction pattern by merging the p1a and the p1b patterns in (*a*). (*c*) Merged diffraction pattern from p2a and p2b on the inner powder ring in (*a*). (*e*) Merged diffraction pattern from p3a and p3b on the inner powder ring in (*d*). (*f*) Merged diffraction pattern from p4a and p4b on the inner powder ring in (*d*).

Figs. 3 ▸(*a*) and 3 ▸(*d*) present typical diffraction patterns after correction, which have a similar hexagonal lattice symmetry as shown in Fig. 1 ▸. The X-ray energy is 416 eV while the Si bandgap is 3.6 eV, which means 1 photon would generate 115.6 electrons. The detector was working at low gain, in which 1 electron provides 1.3 ADUs. Therefore, 1 photon would lead to 150 ADUs. The maximum-intensity peak has around 104.5 ADUs (210 counts) on top of a background of around 200 ADUs (1–2 counts). The dynamic range is around 160, indicating good-quality diffraction images. The beamstop is a B_4_C disc with a diameter of 2 mm, attached to a thin rod and positioned at the center of detector, which was used to block the direct beam. The diffraction peaks fall onto four powder rings visible in total, marked by the white dashed lines. The scattering vector magnitudes of the four powder rings are 0.18 nm^−1^ (Ring 1), 0.31 nm^−1^ (Ring 2), 0.35 nm^−1^ (Ring 3) and 0.47 nm^−1^ (Ring 4). The corresponding *d*-spacings are 34.9 nm, 20.3 nm, 17.9 nm and 13.4 nm according to the relative momentum transfer *Q* of the powder ring.

The index of the peaks can be worked out for this diffraction pattern. Take Ring 1, with a *d*-spacing of 34.9 nm, as the (1, 0) reflection of a perfect close-packed 2D hexagonal lattice, then the (1, 1) peak would have a *d*-spacing of 21.0 nm through calculation of its momentum transfer. Similarly, peak (2, 0) would sit at 17.5 nm and peak (2, 1) at 13.2 nm. These calculated *d*-spacings are close to the measured values as mentioned above. The slight mismatch could arise from two aspects: firstly, the defect density in the (1, 0) and (0, 1) directions could be slightly different, due to symmetry breaking in the self-assembly of the colloid. Secondly, the measurement of the diffraction peak itself could be off-center. In a single-pulse experiment that destroys the part of the sample directly in the beam, there is no option to align scans to guarantee the centering of the sample in the beam. Also, the detector may not be exactly perpendicular to the diffracted beam, rather staying inclined at a small angle. Despite these challenging factors, the mismatch is within 3%, which is safe enough for the peak index calibration.

In Fig. 3 ▸(*a*) on the inner powder ring, there are two sets of diffraction peaks, each with a modulated diffraction pattern surrounding it. The relative angle between the two sets of peaks is 19°. This indicates there were either two colloidal crystals with 19° rotation in orientation, or two subregions of the same crystal, which were illuminated by the same 3 µm X-ray pulse simultaneously. The peaks are far enough apart on the detector that no interference fringes can be seen between them. Each set of peaks comprises six diffraction peaks, showing a general hexagonal symmetry of the lattice. Limited by the X-ray flux, the weaker set of diffraction peaks were not studied further in this work. For the stronger set of diffraction peaks, the opposite (twofold symmetric) diffraction patterns, such as p1a and p1b in Fig. 3 ▸(*a*), have notably similar but 180°-rotated diffraction intensity distributions. These two peaks were overlaid and averaged together, as shown in Fig. 3 ▸(*b*), to have better statistics for reconstruction. The patterns p2a and p2b were also merged to produce the pattern in Fig. 3 ▸(*c*). These two merged peaks are sufficient to map out the 2D displacement distribution and in-plane strain tensor. Similarly, the peaks on the inner powder ring in Fig. 3 ▸(*d*) could also be merged, as shown in Figs. 3 ▸(*e*) and 3 ▸(*f*).

## 4. Phase retrieval and imaging reconstruction

The merged peaks in Figs. 3 ▸(*b*), 3 ▸(*c*), 3 ▸(*e*) and 3 ▸(*f*) were inverted into the real-space images using a Fourier-transform-based phasing algorithm. The well known ‘phase problem’ was solved by making use of the diffraction patterns being ‘oversampled’ and retrieving the missing phase by an iterative phasing method. In iterative phasing, the diffraction pattern was fast Fourier transformed (FFT) repeatedly between reciprocal space and real space, while the measured diffraction data were applied as an amplitude constraint in reciprocal space. In real space, the image was updated on every iteration by setting the amplitude to zero in all regions lying outside a generated physical support, defined as the crystal boundary. When reconstructing the diffraction patterns, the support was updated using the shrink-wrap method (Marchesini, 2007 ▸). Different shrink-wrap thresholds were tried with the best value found at 0.15 of the maximum amplitude. During the iteration loops, the image sharpness was monitored and maximized until a converged reconstruction was reached.

In this work, a combination of error reduction (ER) together with each of difference map, hybrid input–output, relaxed averaged alternating reflection, hybrid projection reflection and averaged successive reflection was tested (Fienup, 1982 ▸; Abrahams & Leslie, 1996 ▸; Luke, 2005 ▸; Bauschke *et al.*, 2002 ▸, 2003 ▸). The reconstructed array contains both amplitude and phase. The reconstructed amplitude defined the crystal boundaries, while the phase is proportional to the displacement field projected onto the corresponding **Q** vector (Robinson & Harder, 2009 ▸). For example, the reconstructed phases of p1 and p2 in Figs. 3 ▸(*b*) and 3 ▸(*c*) would give the displacement field projected on two momentum transfer directions, ϕ_p1_(*x*, *y*) and ϕ_p2_(*x*, *y*). These projected displacement maps were then transformed to Cartesian coordinates. For example, if the directions parallel and perpendicular to the momentum transfer of p1 are set to be the *x* and *y* directions in real space, then the orthogonal in-plane phases, ϕ_*x*_(*x*, *y*) and ϕ_*y*_(*x*, *y*), could be defined as
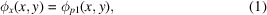

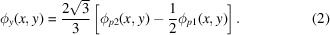
Details of the derivations of equations (1) and (2) can be found in the supporting information.

**Figure 4:**
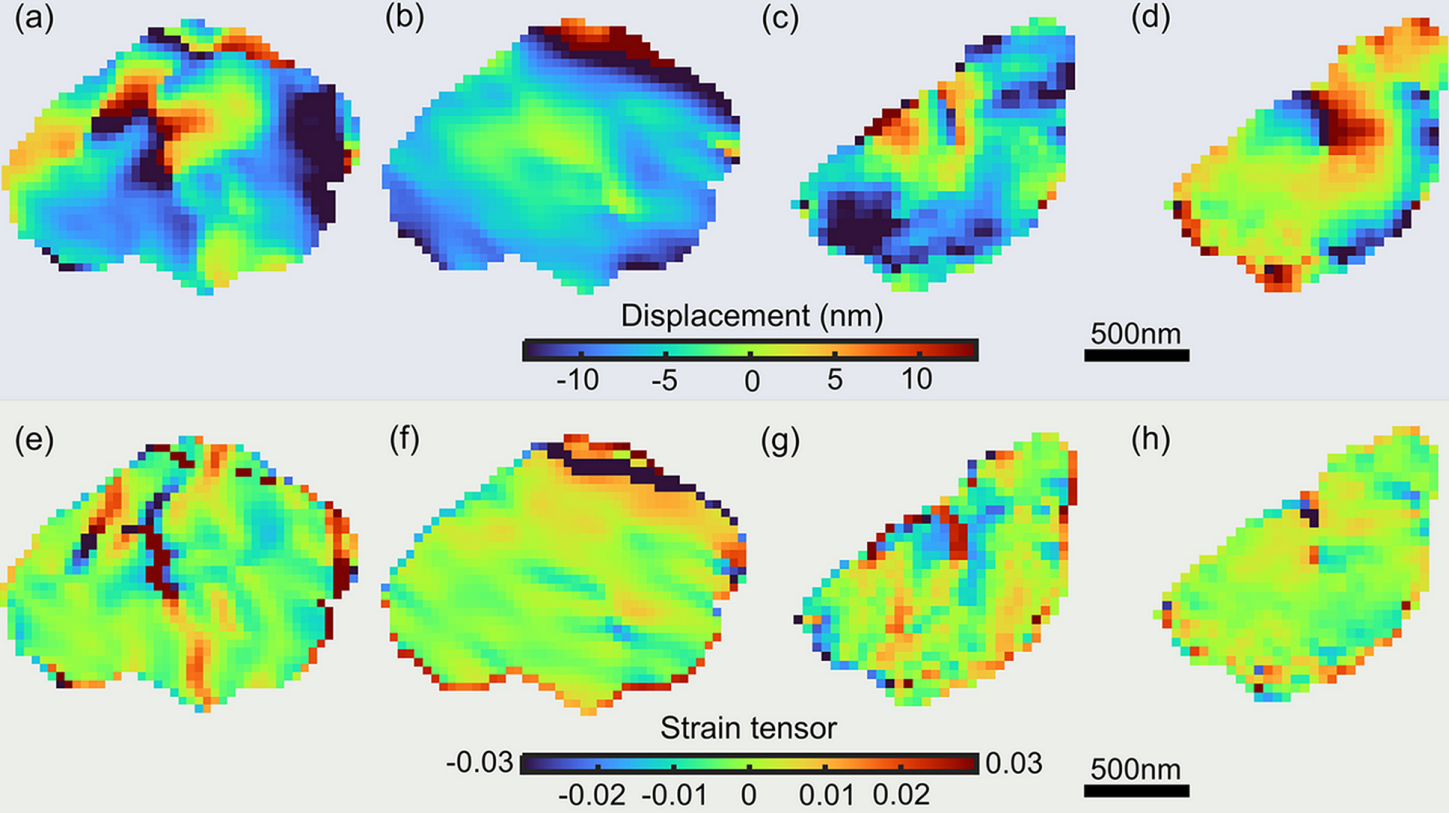
Displacement maps and strain tensor maps of the reconstructed crystals in Figs. 3 ▸(*a*) and 3 ▸(*d*). (*a*) and (*b*) Displacement maps along two orthogonal axes, derived from the reconstruction of peaks in Figs. 3 ▸(*b*) and 3 ▸(*c*). (*c*) and (*d*) Similar displacement maps derived from peaks in Figs. 3 ▸(*e*) and 3(*f*). (*e*)–(*h*) Calculated strain tensor maps from the displacement maps in (*a*)–(*d*), respectively.

Figs. 4 ▸(*a*) and 4 ▸(*b*) show the displacement maps in orthogonal axes of the crystal measured in Fig. 3 ▸(*a*). Similarly, Figs. 4 ▸(*c*) and 4 ▸(*d*) show the orthogonal displacement maps of the crystal in Fig. 3 ▸(*d*). In both cases, displacement variations are scattered over the whole colloid, with displacements up to 15 nm. Sharp changes in the displacement field are also observed inside the crystal, which are the features of crystal defects such as cracks. The strain tensors ɛ_*xx*_ and ɛ_*yy*_, which are the derivatives of the orthogonal displacement along the corresponding axis direction, are calculated and displayed in Figs. 4 ▸(*e*)–4 ▸(*h*). Large strain accumulations are presented near the defect features.

The pixel size of the reconstructed image is dependent on the size of the FFT, which is described as
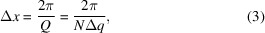
where *Q* is the reciprocal-space vector that covers the whole reciprocal space. Δ*q* is the momentum transfer per pixel, which can be deduced from the angular size per pixel Δθ, 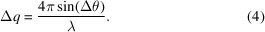
For the crystal in Fig. 4 ▸, the pixel size in the reconstruction is calculated to be 37 nm.

**Figure 5:**
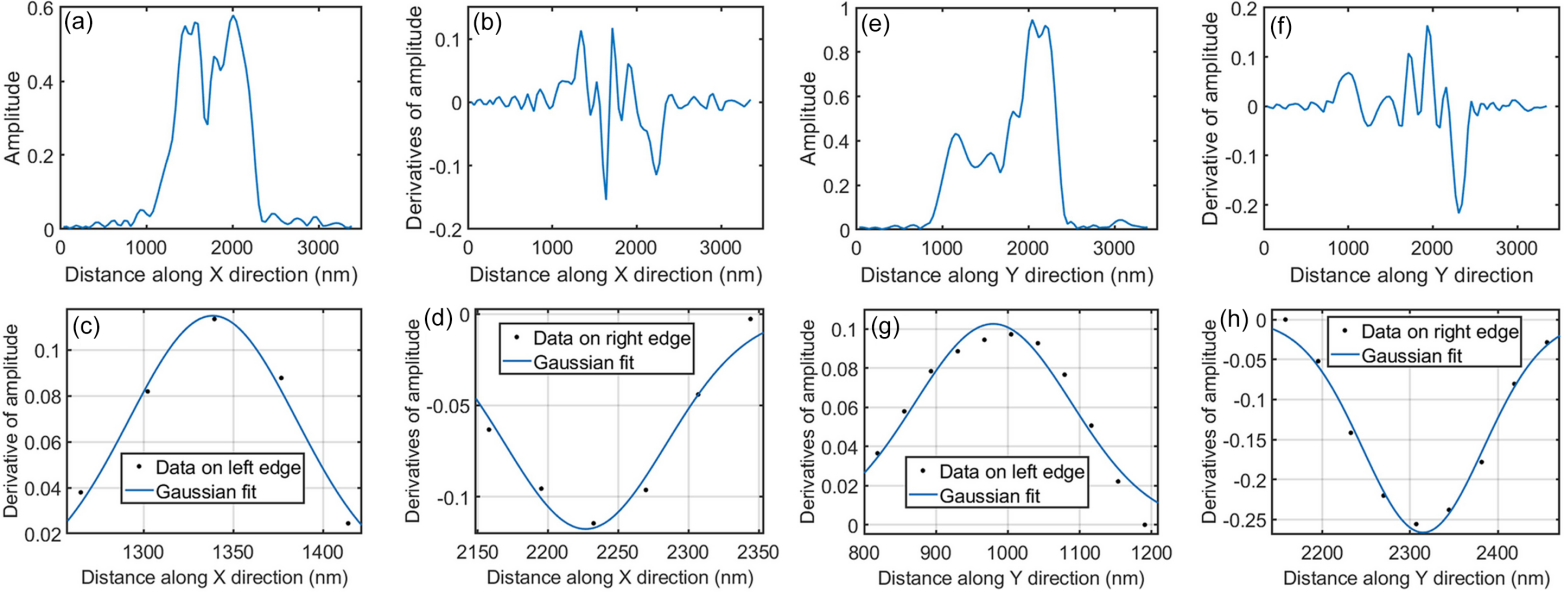
The derivation of resolutions from four edges in the reconstructed crystal in Figs. 4 ▸(*a*) and 4 ▸(*b*). (*a*) and (*e*) Amplitude line plots near the crystal center along the *x* and *y* directions, respectively. (*b*) and (*f*) Corresponding line plots showing the derivative of amplitude over distance. (*c*) and (*d*) Gaussian fits of the left and right edges of the line plot in (*b*). (*g*) and (*h*) Gaussian fits of the left and right edges of line plots in (*f*)

The spatial resolutions of the reconstructed image were calculated from the sharp boundary. Taking the first crystal as an example, Fig. 5 ▸(*a*) presents a horizontal plot of the amplitude near the crystal center. The derivatives of this amplitude plot are shown in Fig. 5 ▸(*b*). The Gaussian fit of the left edge and right edge of the crystal are shown in Figs. 5 ▸(*c*) and 5 ▸(*d*), respectively. The *R*^2^ values of the Gaussian fit are 0.9800 and 0.9690, indicating that the quality of the fitting is good (Table 1 ▸). The calculated spatial resolutions of the *x* direction from the left and right edges are 135 nm and 110 nm, respectively. Similarly, the spatial resolutions along the *y* direction are calculated to be 257 nm and 161 nm.

## 5. Conclusions

A single-shot BCDI experiment has been successfully used to image Au colloidal crystals using an XFEL. Diffraction patterns showing hexagonal symmetry were recorded and their 2D diffraction peaks were indexed. The reconstructions of four of the diffraction patterns show a reproducible crystal shape and present different components of the inner strain field in details. The spatial resolutions of the reconstruction were estimated to be 135 nm and 110 nm horizontally, while in the vertical direction the resolutions are 257 nm and 161 nm. This work opens up the possibility to use a soft XFEL to study the structure of 2D colloidal crystals using BCDI. In the future, a pump laser could be added to explore laser-induced ultrafast melting through direct 2D mapping.

## Supplementary Material

Derivations of equations (1) and (2). DOI: 10.1107/S2052252524012521/it5037sup1.pdf

## Figures and Tables

**Table 1:** The goodness of the Gaussian fit and the calculated image resolution

	Position			
	Horizontal left edge	Horizontal right edge	Vertical left edge	Vertical right edge
σ (nm)	57	47	109	69
*R* ^2^	0.9800	0.9690	0.9502	0.9882
Resolution (nm)	135	110	257	161
