# Supplementary material for: Single-shot X-ray imaging of two-dimensional strain fields in colloidal crystals
Source: IUCrJ. 2025 Feb 11;12(Pt 2):239–44. doi: 10.1107/S2052252524012521 (PMC11878445; doi:10.1107/S2052252524012521)
Supplement: Supplementary file 1 [file m-12-00239-sup1.pdf]

IUCrJ

Volume 12 (2025)

Supporting information for article:

Single-shot X-ray imaging of two-dimensional strain fields in colloidal crystals

Jiacheng Diao, Zichen Gao, Jiadong Fan, Yajun Tong, Hang Ren, Yonggan Nie, Ian Robinson and Huaidong Jiang

1. Derivation of Equation (1) and (2)

Through this experiment, we calculated two phases at two scattering vectors:

$$\phi_{p1}(x,y) = \boldsymbol{\mu}(x,y) \cdot \boldsymbol{q}_{p1}$$

$$\phi_{p2}(x,y) = \boldsymbol{\mu}(x,y) \cdot \boldsymbol{q}_{p2}$$

We can define a new orthogonal axis:

$$\boldsymbol{q}_{p1} = (\boldsymbol{q}_{p1} \cdot \boldsymbol{q}_x)\boldsymbol{q}_x + (\boldsymbol{q}_{p1} \cdot \boldsymbol{q}_y)\boldsymbol{q}_y$$

$$\boldsymbol{q}_{p2} = (\boldsymbol{q}_{p2} \cdot \boldsymbol{q}_x)\boldsymbol{q}_x + (\boldsymbol{q}_{p2} \cdot \boldsymbol{q}_y)\boldsymbol{q}_y$$

In this case, we make the $\boldsymbol{q}_{p1}$ parallel to $\boldsymbol{q}_x$ as followed:

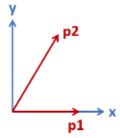

Therefore,

$$\boldsymbol{q}_{p1} = \boldsymbol{q}_x$$

$$\boldsymbol{q}_{p2} = \frac{1}{2}\boldsymbol{q}_x + \frac{\sqrt{3}}{2}\boldsymbol{q}_y$$

In such case, the phase on the orthogonal axis can be derived from the calculated two phases above:

$$\phi_x(x,y) = \phi_{p1}(x,y)$$

$$\phi_y(x,y) = \frac{2\sqrt{3}}{3}\left(\phi_{p2}(x,y) - \frac{1}{2}\phi_{p1}(x,y)\right)$$
